# Reproducibility of Retinal Nerve Fiber Layer Measurements with Manual and Automated Centration in Healthy Subjects Using Spectralis Spectral-Domain Optical Coherence Tomography

**DOI:** 10.5402/2012/860819

**Published:** 2012-07-30

**Authors:** Alex P. Lange, Reza Sadjadi, Fiona Costello, Ivo Guber, Anthony L. Traboulsee

**Affiliations:** ^1^Department of Ophthalmology and MS Clinic, Department of Neurology, University of British Columbia, Vancouver, BC, Canada V5Z 3N9; ^2^MS Clinic, Department of Neurology, The University of British Columbia, Vancouer, BC, Canada V6T 2B5; ^3^Departments of Clinical Neurosciences and Surgery, Hotchkiss Brain Institute, University of Calgary, Calgary, AB, Canada T2N 1N4; ^4^Jules-Gonin Eye Hospital, University of Lausanne, 1015 Lausanne, Switzerland

## Abstract

*Objective*. The aim of this study was to test the reproducibility of the Heidelberg Spectralis SD-OCT and to determine if provided software retest function for follow-up exam is superior to manual centration. *Design*. Prospective, cross-sectional study. *Participants*. 20 healthy subjects. *Methods*. All subjects underwent SD-OCT testing to determine retinal nerve fiber layer (RNFL) measurements sequentially on two different days and with two different centration techniques. Within-subject standard deviation, coefficient of variation, and intraclass correlation coefficient were used to assess reproducibility. *Results*. RNFL measurements showed high reproducibility, low within-subject standard deviation (1.3), low coefficient of variation (0.63%), and low intra-class correlation coefficient (0.98 (95% CI 0.97–0.99)) in the automated centration and manual centration groups for average RNFL Thickness. Quadrants showed slightly higher variability in the manual group compared to the automated group (within-subject standard deviation 2.5–5.3 versus 1.1–2.4, resp.). *Conclusions*. SD-OCT provides high-resolution RNFL measurements with high reproducibility and low variability. The re-test function allows for easier recentration for longitudinal examinations with similar results in average RNFL, but less variability in quadrant RNFL. SD-OCT high reproducibility and low variability is a promising fact and should be further evaluated in longitudinal studies of RNFL.

## 1. Introduction

Optical coherence tomography (OCT) is a noninvasive technique that provides high-resolution, cross-sectional tomographic imaging of retinal tissue using backscattered light. OCT imaging is analogous to ultrasound B-mode imaging but uses infrared light instead of ultrasound waves. From multiple axial scans (A scans) at different transverse locations, a two-dimensional, cross-sectional image can be obtained [1].

Until recently, third-generation time-domain OCT (TD-OCT) using Stratus OCT (Carl Zeiss Meditec AG, Jena, Germany) has been widely used to acquire images at a rate of 400 axial scans per second with an axial resolution of 10 *μ*m [2]. The recently introduced fourth-generation, spectral-domain OCT (SD-OCT) has improved depth resolution by a factor of three (axial resolution up to 3.8 *μ*m) and allows a significantly higher acquisition speed (40,000 axial scans per second) resulting in improved image quality and minimized motion artefacts [3]. Furthermore, software improvements allow reconstruction of a three-dimensional image of the retina with SD-OCT.

Recent studies have shown differences between other SD-OCT machines (Cirrus SD-OCT, Carl Zeiss Meditec AG, Jena, Germany [4–9], RTVue-100, Optovue Inc., Fremont, CA, USA [10] and Spectralis, Heidelberg Engineering, Heidelberg, Germany [11, 12]) and TD-OCT in healthy controls and glaucoma patients. These studies showed better reproducibility compared to TD-OCT and significant differences in retinal nerve fiber layer thickness (RNFLT) measurements between the two generations of OCT machines.

Heidelberg Spectralis OCT software helps centering the optic disc on a frozen fundus image and uses an integrated eye tracking system to compensate for eye movement artefacts during data acquisition. Additionally, Heidelberg noise reduction technology (ART) improves image quality by averaging several consecutive scans and increasing signal-to-noise ratio [13]. Furthermore, software-assisted re-test function, using previous landmarks (location and direction of blood vessels) to recenter the SD-OCT beam to a previously scanned location, facilitates follow-up exams in longitudinal studies. 

This would theoretically help to rescan the same location with every follow-up examination without the risk of variability due to different locations. The potential value of this re-centration function is significant to future longitudinal studies as it allows to detect very small changes in RNFL over time and can distinguish these changes from test-retest variability inherent to the technology.

However, before this new technology can be used as outcome measure for longitudinal study of slow, subtle axonal loss (e.g., in MS or glaucoma), it is crucial to determine the reproducibility and variability. 

Therefore, the aim of this study is to determine reproducibility and variability of RFNL measurements using Spectralis SD-OCT with optimized settings and to compare manual centration versus automated re-test function in follow-up examinations of healthy controls.

## 2. Materials and Methods 

### 2.1. Recruitment of Study Subjects

Healthy subjects were recruited from hospital staff by advertisement. Subjects with eye or systemic diseases by history were not enrolled in this study.

### 2.2. Spectral-Domain Optical Coherence Tomography

**Figure 1:**
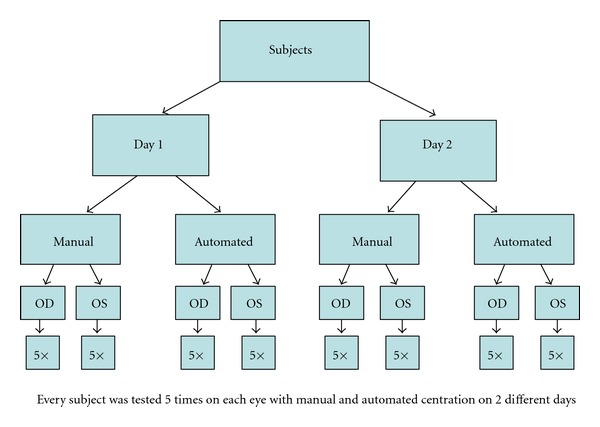
OCT algorithm for every included subject.

Heidelberg Spectralis OCT (software version 5.1.2, Heidelberg Engineering, Heidelberg, Germany) with RNFL protocol in high-resolution mode (resolution 3.8 *μ*m, 19,000 scans per second) was used. Sixteen consecutive circular B Scans with a diameter of 3.4 mm were automatically averaged to reduce speckle noise (ART mode 16). The online tracking system compensated for eye movements. Both eyes of every subject were measured several times and the best centered scan with the highest quality score (≥25) [14] was chosen as a reference scan. Every eye was measured several times with manual centration (the circle scan was manually centered on the frozen fundus image) and automated re-test function (the previously taken reference scan was selected and the circle scan was positioned by the software) without pupil dilation [15]. When 5 scans (including reference scan) with a quality score of ≥25 were achieved, the exam was stopped and images were taken for analysis. The same procedure was performed on a second day within one week.  Figure 1 shows the exact OCT algorithm. 

### 2.3. Statistical Analysis

Microsoft Office 2007 and SPSS Version 16.0 were used to explore descriptive analysis and mean comparisons (Student's *t*-test). The data was tested for normality. SD-OCT reproducibility was assessed using within-subject standard deviation (Sw; within subject standard deviation is the square root of the within-subjects sum of squares divided by degree of freedom), coefficient of variation (CV; within-subject standard deviation divided by the arithmetic mean, expressed as a percentage with CV <5% as good reproducibility), and intraclass correlation coefficient (ICC).

### 2.4. Ethics Statement

This study was reviewed by Clinical Research Ethics Board at the University of British Columbia and received research ethical approval prior to recruitment. A study information sheet was provided and informed consent was obtained prior to enrolment in the study. 

## 3. Results

39 eyes of 20 healthy controls were included in the study. One eye had to be excluded due to inability of the OCT to recognize RNFL margins in a patient with high myopia. Theoretically, the software allowed for manual correction, but we did not want to bias any scans. There were 11 male and 9 female subjects with mean age 31.51 years (SD = 8.04 years). Mean RNFL value was 98.46 *μ*m (95% CI 97.57–99.35 *μ*m) for all eyes. An overview on descriptive data is given in Table 1.

In total, 3800 values for RNFL were included into statistical analysis. The data was normally distributed (Shapiro-Wilk > 0.05). The Sw, CV, and ICC findings are shown in Table 2. 

In general, the overall reproducibility (day 1 + day 2) was excellent in both groups, especially for average RNFL with ICC of 0.98 (95% CI 0.974–0.990) in the automated group and ICC of 0.99 (95% CI 0.998–0.999) in the manual group. The quadrants showed slightly higher variability with Sw between 2.5 and 5.3 in the manual and Sw between 1.1 and 2.4 in the automated centration group.

If analysis was performed on data of day 1 and 2 separately (intersession reproducibility), no significant difference was found (*P* = 0.38; item reliability Cronbach's alpha = 0.99).

If analysis was performed on data of right and left eyes separately, no significant difference was found (*P* = 0.19; item reliability Cronbach's alpha = 0.98).

## 4. Discussion

This study evaluated reproducibility in the fourth-generation Heidelberg Spectralis OCT with optimum settings and compared manual centration and automated centration. 

We were able to demonstrate high reproducibility of RNFL measurements with high ICC of 0.98 (95% CI 0.97–0.99) and low CV measuring less than 1%. Our results show less variability compared to those of Langenegger et al. [14], who in a cross-sectional study of 56 healthy controls and 47 glaucoma eyes using eye-tracker showed CV between 2.7% and 10.5% in the manual group and 1.3%–3.5% in the re-test function group. Our slightly lower variability can be explained by the high-resolution mode, allowing an axial resolution of 3.8 *μ*m, whereas their high-speed mode cuts the resolution in half, and by using the eye tracker in our manual centration group as well, facilitating the centration. On the other hand, a cross-sectional study performed by Serbecic et al. [15] on both eyes of 31 healthy participants with activated re-test function found a similar CV between 0.29% and 1.07% and did not find any differences between measurements in high-speed or high-resolution mode. This implicates that the eye tracker rather than the higher resolution can explain these differences.

 Another cross-sectional study on 30 healthy eyes that used no re-test function reported lower reproducibility with eyes ICC of 0.96 (95% CI 0.94–0.99) for average RNFLT and ICC between 0.87 and 0.96 for quadrants with Spectralis SD-OCT [12]. Although not directly comparable, especially quadrants variability was lower in our study when the re-test function was used.

In contrast, Seibold et al. [11] in a study on 80 healthy eyes with the same device showed ICC of 0.90 (95% CI 0.88–0.95), which is significantly lower than our results. They used only a single scan and compared it to a second single scan within 8 weeks using the follow-up function. The longer interval and the lack of repeated scanning might explain the differences. 

On the other hand, previously published studies using the older generation TD-OCT devices showed lower ICC between 0.83 [2] and 0.95 [16] (Table 3).

A coefficient of variation of less than 1% in RNFL measurements of up to 140 *μ*m will give a reproducibility within ±1-2 *μ*m. This is the maximum that could be expected from the axial resolution of 3.8 *μ*m of this device and superior to the older technology with axial resolution of 10 *μ*m. One study found a difference in thickness of 8 micrometer to be within normal test-retest variability of the older TD-OCT machine [17].

The high reproducibility of the SD-OCT technology is very important in two particular applications: long-term follow-up of MS patients and monitoring of glaucoma progression.

In a recent cross-sectional study, RNFL measurements in healthy controls showed an age-related decrease of 2 *μ*m per decade. Patients with relapsing-remitting MS (RRMS) in the interval were shown in a longitudinal study to have an average decrease in RNFL of 2 *μ*m per year, which was a tenfold increase relative to healthy controls [18]. This was found using the older generation TD-OCT, and the results were only significant in the groups with more than 3 years of follow-up. 

Glaucoma patients have been shown to have a median decrease in RNFL thickness measuring 3.3 *μ*m per year [19]. Considering the recent launch of the commercially available SD-OCT and the slowly progressive character of glaucoma and MS, we must wait for more longitudinal SD-OCT data to become available. But our data show that reproducibility of the used machine is high enough to detect very small increments of change in RNFLT over time and, moreover, can distinguish these changes from test-retest variability inherent to the technology.

The Heidelberg Spectralis SD-OCT software has the advantage of providing automated re-test in follow-up examinations. We were able to show that manual centration and automated re-test function were equally reliable measuring the average RNFLT. However, there was a compensated variability in manual measurements compared to the automated ones. The most likely explanation is a possible decentration during manual scans. RNFL has been shown to decrease in more peripheral zones of the optic disc [20, 21]. While the average value for one shifted quadrant may decrease as a result of decentration, a corresponding quadrant value increases as there is more central area covered by this quadrant. This would also explain why average RNFL thickness is not affected. The automated centration seems to be more reliable if individual quadrant values are investigated as independent outcome measures, which is particularly important in the follow-up of glaucoma patient, where localised thinning of RNFL is expected.

Although we did not find any significant difference in reproducibility of average RNFLT between centration techniques, automated re-test function is a better outcome measure in clinical trials and research studies as it is a more standardised method and less subject to human error.

Shortcomings of our study were that only healthy eyes with generally higher RFNL values than glaucoma or MS eyes were included and we were not able to demonstrate the variability in thinner RNFL. On the other hand, RNFL analysis in different quadrants covered reproducibility testing within a range of 70 to 125 *μ*m comparable to RNFL thickness measured in MS and glaucoma eyes [18, 19]. The second point is that inclusion of both eyes of the subjects may produce artificial inflation of study power and falsely narrow confidence intervals. The third point is that only well-centered scans were taken into analysis and scans were all performed by the same experienced operator. We are therefore not able to demonstrate variability in off-centered scans or interoperator variability. 

In conclusion, SD-OCT machine shows high reproducibility in measuring RNFLT in healthy controls and may prove to be a valuable tool for assessing small longitudinal changes in RNFLT. Manual and automated centration showed similar reproducibility for average RNFL, with slightly more variation in the manual group for quadrants suggesting a use of the automated re-test function if quadrants are the primary outcome measure as in glaucoma monitoring.

## Figures and Tables

**Table 1:** Descriptive data of study subjects.

Number of subjects	20
Males	11
Females	9
Number of eyes included	39
Mean age (years, ± SD)	31.51 (8.04)
Number of emmetropic eyes	24
Number of myopic eyes	15
Mean RNFL in *μ*m (95% CI)	98.46 (97.57–99.35)

**Table 2:** SD-OCT RNFL thickness reproducibility.

	Manual centration				Automatic centration			
	Mean RNFL in *μ*m (95% CI)	Sw	CV %	ICC (95% CI)	Mean RNFL in *μ*m (95% CI)	Sw	CV %	ICC (95% CI)
Average	98.35 (97.07–99.62)	1.6	0.65	0.999 (0.998–0.999)	98.57 (97.33–99.81)	1.3	0.63	0.983 (0.974–0.990)
Temporal	76.06 (74.59–77.53)	2.8	0.97	0.980 (0.970–0.988)	74.83 (73.54–76.11)	1.1	0.86	0.998 (0.998–0.999)
Superior	118.35 (116.36–120.31)	5.3	0.83	0.997 (0.995–0.998)	118.84 (116.95–120.73)	2.1	0.80	0.999 (0.983–0.999)
Nasal	71.80 (69.75–73.85)	3.2	1.43	0.989 (0.984–0.994)	72.86 (70.98–74.75)	2.4	1.29	0.998 (0.998–0.999)
Inferior	126.87 (124.83–128.91)	2.5	0.80	0.997 (0.995–0.998)	127.31 (125.35–129.27)	1.9	0.77	0.999 (0.999–0.999)

SD-OCT: spectral-domain optical coherence tomography; RNFL: retinal nerve fiber layer; CI: confidence intervals; Sw: within subject standard deviation; CV: coefficient of variation; ICC: intraclass correlation.

**Table 3:** Overview published studies comparing RNFLT in SD-OCT.

Author	SD-OCT used	Study population (eyes)	Results
Leung et al. [6]	Cirrus and stratus	83 glaucoma 97 controls	Cirrus: (95% CI) (i) repeatability 5.12 (3.87–6.37) (ii) CV 1.89% (1.43–2.36) (iii) ICC 0.975 (0.945–0.991) Stratus: (i) repeatability 11.10 (8.34–13.87) (ii) CV 3.58% (2.69–4.48) (iii) ICC 0.866 (0.741–0.933)

Vizzeri et al. [8]	Cirrus	78 glaucoma 32 controls	Cirrus (95% CI) (i) Sw 1.3 (ii) CV 1.5% (iii) ICC 0.96 (0.94–0.98)

Kim et al. [9]	Cirrus and stratus	27 controls	Cirrus (95% CI) ICC 0.984 Stratus ICC 0.894

Gonzalez-Garcia et al. [10]	RTVue-100	76 glaucoma 60 controls	RTVue (95% CI) (i) Sw 0.01 (ii) CV 0.27% (iii) ICC 0.99 (0.98–0.99)

Seibold et al. [11]	Cirrus, Spectralis, RTVue-100, stratus	80 controls	Cirrus (95% CI) (i) Reproducibility 8.89 (5.75–12.02) (ii) CV 3.03% (1.47–4.03) (iii) ICC 0.92 (0.87–0.97) Spectralis (i) Reproducibility 11.72 (9.31–14.13) (ii) CV 3.91% (2.81–4.77) (iii) ICC 0.90 (0.85–0.95) RTVue-100 (i) Reproducibility 6.59 (5.59–7.59) (ii) CV 2.09% (1.71–2.41) (iii)ICC 0.97 (0.96 to 0.98) Stratus (i) Reproducibility 8.83 (7.21–10.45) (ii) CV 2.86% (2.17–3.42) (iii) ICC 0.94 (0.91 to 0.97)

Arthur et al. [12]	Spectralis	30 controls	Spectralis (95% CI) ICC 0.96 (0.94–0.99) Stratus ICC 0.86 (0.77–0.94)

Langenegger et al. [14]	Spectralis	56 controls 47 glaucoma	Spectralis (95% CI) (i) CV 1.0% with retest function (ii) CV 1.6% without retest function (iii) ICC 0.99 (0.98)

Serbecic et al. [15]	Spectralis	62 Controls	Spectralis (95% CI) CV 0.545–3.97%

Current study	Spectralis	39 controls	Spectralis (95% CI) (i) Reproducibility 1.30 (ii) CV 0.63% (iii) ICC 0.983 (0.97–0.99)
